# Magnetic nanoparticles for biomedical NMR-based diagnostics

**DOI:** 10.3762/bjnano.1.17

**Published:** 2010-12-16

**Authors:** Huilin Shao, Tae-Jong Yoon, Monty Liong, Ralph Weissleder, Hakho Lee

**Affiliations:** 1 Center for Systems Biology, Massachusetts General Hospital, 185 Cambridge St, CPZN 5206, Boston, MA 02114, U.S.A.; 2 Department of Applied Bioscience, CHA University, Seoul 135-081, Korea; 3 Department of Systems Biology, Harvard Medical School, 200 Longwood Av, Alpert 536, Boston, MA 02115, U.S.A.

**Keywords:** biosensor, diagnostics, magnetic nanoparticle, microfluidics, nuclear magnetic resonance

## Abstract

Rapid and accurate measurements of protein biomarkers, pathogens and cells in biological samples could provide useful information for early disease diagnosis, treatment monitoring, and design of personalized medicine. In general, biological samples have only negligible magnetic susceptibility. Thus, using magnetic nanoparticles for biosensing not only enhances sensitivity but also effectively reduces sample preparation needs. This review focuses on the use of magnetic nanoparticles for in vitro detection of biomolecules and cells based on magnetic resonance effects. This detection platform, termed diagnostic magnetic resonance (DMR), exploits magnetic nanoparticles as proximity sensors, which modulate the spin–spin relaxation time of water molecules surrounding molecularly-targeted nanoparticles. By developing more effective magnetic nanoparticle biosensors, DMR detection limits for various target moieties have been considerably improved over the last few years. Already, a library of magnetic nanoparticles has been developed, in which a wide range of targets, including DNA/mRNA, proteins, small molecules/drugs, bacteria, and tumor cells, have been quantified. More recently, the capabilities of DMR technology have been further advanced with new developments such as miniaturized nuclear magnetic resonance detectors, better magnetic nanoparticles and novel conjugational methods. These developments have enabled parallel and sensitive measurements to be made from small volume samples. Thus, the DMR technology is a highly attractive platform for portable, low-cost, and efficient biomolecular detection within a biomedical setting.

## Introduction

Rapid and sensitive measurement of clinically relevant biomarkers, pathogens and cells in biological samples would be invaluable for disease diagnosis, monitoring of malignancy, and for evaluating therapy efficacy in personalized medicine. To translate such molecular measurements into clinical settings, however, an assay would need to 1) provide high sensitivity and specificity, 2) minimize sample preparation and sample volume, and 3) ideally allow concurrent detection of diverse target moieties through multiplexed measurements. Biosensing strategies based on magnetic nanoparticles (MNPs) have recently received considerable attention, since they offer unique advantages over traditional detection methods. Specifically, because biological samples exhibit negligible magnetic background, MNPs can be used to obtain highly sensitive measurements in turbid samples with reduced sample preparation. In contrast, traditional detection strategies based on optical techniques, for example, are often affected by scattering, absorption, autofluorescence, and require extensive sample purification before measurements can be made.

To detect biomarkers using MNPs, several technologies have been developed [1]. These include techniques that use magnetometers, such as superconducting quantum interference device (SQUID) [2–4], magnetoresistive sensors [5–11], and Hall sensors [12], which directly measure the magnetic fields from magnetically-labeled biological targets. Another technology that has achieved considerable success is diagnostic magnetic resonance (DMR). Based on nuclear magnetic resonance (NMR) as the detection mechanism, DMR exploits MNPs as proximity sensors, which modulate the spin–spin relaxation time of water molecules adjacent to the molecularly-targeted MNPs. The latter create a local magnetic field and induce a change in proton relaxation rate in billions of neighboring water molecules [13]. Direct detection of magnetic moments with magnetometers requires MNP-labeled targets to be closely positioned to the sensing elements. DMR assays, however, are faster and simpler since the analytical signal is generated from the entire sample volume.

By developing optimized MNPs, DMR detection sensitivities for various target moieties have been considerably improved. To date, numerous magnetic biosensors have been designed to identify and quantify a wide range of targets including DNA/mRNA, proteins, small molecules/drugs, bacteria, and tumor cells. More recently, the development of miniaturized, chip-based NMR detector systems has served to further enhance DMR technology [14–16]. Such detectors can perform highly sensitive measurements on microliter sample volumes and in a multiplexed format. With the integration of key components (i.e., microcoils, microfluidic networks, NMR electronics, and a portable magnet), the DMR systems have now demonstrated their potential for portable, sensitive and rapid operation in a point-of-care setting [14,17–19].

This review will report on various aspects of MNPs, their use in DMR sensing, assay modes, and on recent developments in improving detection sensitivities. Specific biomedical DMR applications will also be summarized.

## Magnetic nanoparticles and their relaxation properties

Nanoparticles have tremendous potential in the field of biomedical applications, primarily on account of their similar size to biological molecules, and because their properties can be fine-tuned during chemical synthesis. In particular, MNPs can be synthesized in such a way as to possess unique superparamagnetic properties, to be biocompatible, and to remain inert with respect to cells and molecules of interest. As the size of magnetic objects shrinks to the nanometer scale, it becomes energetically more favorable for them to have a single magnetic domain than to form domain walls and a consequent multi-domain structure [20]. The upper limit for a single domain [~(*A*/2*K*)^1/2^] is determined by the material properties: the exchange stiffness (*A*) and the anisotropy constant (*K*). For most magnetic materials (e.g., ferrite and iron), MNPs with a diameter <20 nm will have a single domain with magnetic moments aligned in a particular direction defined by magnetic anisotropy. At sufficiently high temperatures (above blocking temperature), thermal energy can induce free rotation of the magnetic moment. Thus, when MNPs are grouped together, they display a form of paramagnetic behavior, known as superparamagnetism: MNPs assume overall magnetic moments when placed in an external magnetic field but lose their moments when the field is removed. Distinct from paramagnetism, which arises from individual spins at the atomic or molecular level, superparamagnetism applies to magnetic elements that already assume a magnetically-ordered spin state (typically ferromagnetic or ferrimagnetic). This superparamagnetic property enables MNPs to avoid spontaneous aggregation in solution, a feature that makes them suitable for many biomedical applications. In its simplest form, an MNP is comprised of an inorganic magnetic core and a biocompatible surface coating that stabilizes the particle in physiological conditions. By applying suitable surface chemistry, functional ligands can be integrated and confer the MNP with molecular specificity.

### Synthesis of magnetic nanoparticles

Synthetic methods for MNPs have been recently reviewed [15–16,21–25]. A variety of chemical methods, ranging from traditional wet chemistry to high-temperature thermal decomposition, have been employed to synthesize MNPs. Colloidal iron oxide nanoparticles, which are used as clinical magnetic resonance imaging (MRI) contrast agents, are generally prepared via an aqueous co-precipitation method [25–26]. During these hydrolytic processes, control of solution pH and the addition of suitable coating surfactants are critical for regulating the nanoparticle size as well as the magnetic properties. Unfortunately, depending on the synthesis procedure used, magnetization can vary significantly among nanoparticles of similar sizes.

More recently, high quality MNPs have been prepared through thermal decomposition of organometallic precursors, in nonhydrolytic organic solutions containing surfactants [15–16,27–29]. Monomers are generated via high-temperature thermal decomposition of precursors. Above a supersaturation level, these monomers then aggregate to induce nucleation and nanoparticle growth. By tuning the growth conditions during this procedure (such as precursor choice, monomer concentration, growth temperature and time), it is possible to control the size, composition, and crystallinity of the nanoparticles. While high-temperature decomposition markedly improves size control, size distribution and crystallinity of MNPs, the resulting particles are encased in a hydrophobic coating. In order to achieve nanoparticle stability in aqueous media, this approach requires additional modifications. Techniques, such as the addition of an amphiphilic polymer [30] or surfactant exchange strategies [31–33], have been examined for their ability to transfer the hydrophobic MNPs into the aqueous phase.

### Magnetic relaxation mechanism

When placed in an external field, each MNP creates a local magnetic field, which increases the field inhomogeneity. When water molecules diffuse within the periphery of the MNPs, the coherent precessions of water proton spins are perturbed. The net effect is a change in the magnetic resonance signal, which is measured as a shortening of the longitudinal (*T*_1_, spin–lattice) and transverse (*T*_2_, spin–spin) relaxation times. The capacities of MNPs to decrease *T*_2_ and *T*_1_ are respectively defined as the transverse (*r*_2_) and the longitudinal (*r*_1_) relaxivities. Typically, because the transverse relaxivities (*r*_2_) of MNPs are greater than their longitudinal relaxivities (*r*_1_), *T*_2_ is used for NMR-based biosensing applications. With a higher *r*_2_ relaxivity, fewer numbers of nanoparticles are required to produce detectable *T*_2_ changes.

Within an ensemble of MNPs, magnetic relaxation properties depend on more than simply the particles’ relaxivities; the organizational state of the ensemble is also important. Unlike evenly dispersed MNPs, aggregates of nanoparticles (self-assembled magnetic clusters) have been shown to enhance the net rate of transverse relaxation [13,34]. This unique phenomenon, known as magnetic relaxation switching (MRSw), is a cooperative process in which the interacting nanoparticles become more efficient at dephasing the spins of neighboring water protons, leading to a decrease in *T*_2_ relaxation time. The phenomenon can be explained by the outer-sphere theory. For a given volume fraction of MNPs in solution, *T*_2_ of the sample is inversely proportional to the cross-sectional area of the particles [35–36]. Thus, the same amount of magnetized material is much more effective when dispersed as fewer large nanoparticles than as a greater number of smaller ones [35]. In MRSw, nanoparticles aggregate to form self-assembled clusters, and the consequent increase in cross-sectional area of the particles shortens *T*_2_ relaxation times.

### DMR assay configurations

Analogous to MRI, DMR exploits targeted MNPs to modulate the spin–spin *T*_2_ relaxation time of biological samples. Depending on the size of the target biomarker, DMR assays can take two forms.

**Figure 1:**
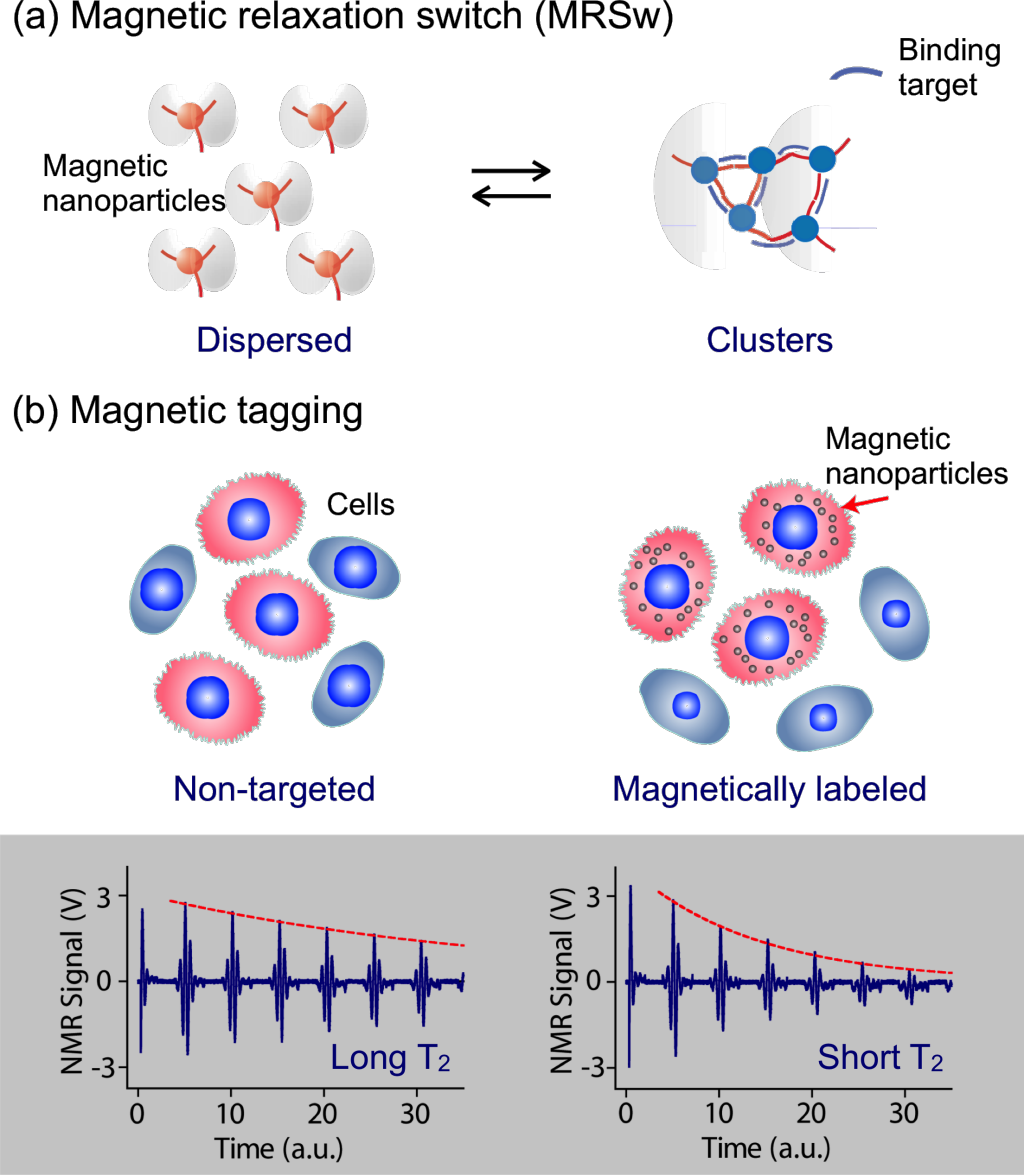
DMR assay configurations with magnetic nanoparticles (MNPs). (a) Magnetic relaxation switching (MRSw) assays detect the clustering of MNPs (forward switching), using a small target biomarker as a cross-linker, or the disassembly of pre-formed clusters (reverse switching) using an enzyme or competitive binding. When dispersed MNPs aggregate upon binding to targets, the self-assembled magnetic clusters become more efficient at dephasing nuclear spins of surrounding water protons, leading to a decrease in *T*_2_ relaxation time. The reverse is true upon cluster disassembly. (b) Magnetic tagging assays detect the presence of bound MNPs on larger biological entities. Tagging of cell surface markers via targeted MNPs imparts a magnetic moment to cells, leading to a decrease in *T*_2_ relaxation time. Unbound MNPs must be removed to ensure detection sensitivity of this assay mode. (Reproduced with permission from [13–14]. Copyright 2002, 2008 Nature Publishing Group.)

For small molecular targets with sizes less than or comparable to that of the MNPs, MRSw assays can be used effectively for their detection and quantification. Small molecular analytes, such as drugs, metabolites, oligonucleotides, and proteins, can cross-link MNPs to promote relaxation switching. As indicated in Figure 1a, MRSw assays can be designed to cause forward switching: a process whereby molecular targets are used as cross-linking agents to assemble MNPs into clusters, thus effecting a corresponding decrease in *T*_2_. Alternatively, the assays can cause reverse switching, where enzymatic cleavage or competitive binding of molecular targets disassembles pre-formed clusters to cause an increase in *T*_2_. Note that MRSw assays are carried out without removing excess unbound MNPs.

For larger biological structures such as bacteria, entire mammalian cells or cellular components, targeted MNPs can be used to tag cell surface markers to impart a magnetic moment (Figure 1b). The change of 1/*T*_2_ is proportional to the number of MNPs bound, and also indicative of the abundance of relevant surface biomarkers. Unlike MRSw assays, this magnetic tagging strategy requires washing steps to remove excess unbound MNPs from the tagged biological targets.

## Optimal magnetic nanoparticles for DMR detection sensitivity

To enhance DMR detection sensitivity, MNPs should possess the following characteristics: 1) exhibit superparamagnetic properties; 2) have high stability in aqueous media to avoid spontaneous aggregation, which could mimic target-induced clustering; 3) have high magnetization and transverse relaxivity (*r*_2_) to induce pronounced *T*_2_ changes; and 4) have good surface chemistry to simplify conjugational procedures for attaching affinity molecules, such as antibodies and peptides. The MNPs and their representative strategies described below have been shown to be uniquely suited for DMR applications.

### Cross-linked iron oxide nanoparticles

Cross-linked iron oxide (CLIO) nanoparticles have been widely used for DMR applications on account of their excellent stability and biocompatibility [13,37–42]. CLIO nanoparticles contain a superparamagnetic iron oxide core (3–5 nm monocrystalline iron oxide) composed of ferrimagnetic magnetite (Fe_3_O_4_) and/or maghemite (γ-Fe_2_O_3_). The metallic core is subsequently coated with biocompatible dextran, before being cross-linked with epichlorohydrin and activated by ammonia to provide primary amine group functionality. The amine groups can then be easily reacted with various agents containing anhydride, hydroxyl, carboxyl, thiol, or epoxide groups, to confer molecular specificity to the nanoparticle through bioconjugation [43]. Amine-terminated CLIO nanoparticles have an average hydrodynamic diameter of 25–40 nm, approximately 40–80 amines per nanoparticle for bioconjugation, and a *r*_2_ of ~50 s^−1^·mM^−1^ [Fe] [13,44]. Despite their relatively low *r*_2_, their unique coating makes CLIO nanoparticles exceedingly robust for use in biological applications.

### Doped-ferrite nanoparticles

The magnetization of ferrite nanoparticles can be further enhanced by doping the ferrite with ferromagnetic elements such as manganese (Mn), cobalt (Co) or nickel (Ni) [23,27,45]. Among the singly-doped ferrite MNPs, MnFe_2_O_4_ nanoparticles were found to exhibit the highest magnetization and *r*_2_ value, on account of their electron spin configurations, followed by FeFe_2_O_4_, CoFe_2_O_4_, and NiFe_2_O_4_. More recently, it has been demonstrated that magnetization can be further enhanced via additional Zn^2+^ dopant control in MnFe_2_O_4_ nanoparticles [46]. In addition, nanoparticle magnetization is known to increase with particle size [33]. Ideally, each magnetic spin within a bulk magnetic material would be aligned parallel to the external magnetic field. However, in the nanoscale regime, surface spins tend to be tilted, a feature that reduces the overall magnetic moment. By increasing the MNP size, this surface effect is decreased, which in turn increases the magnetization. It has also been noted that transverse relaxivity *r*_2_ is proportional to the cross-sectional area of the magnetic core [36]. Thus, increasing MNP size is an efficient method for enhancing *r*_2_, since this strategy increases both the magnetization as well as the particle cross-sectional area.

**Figure 2:**
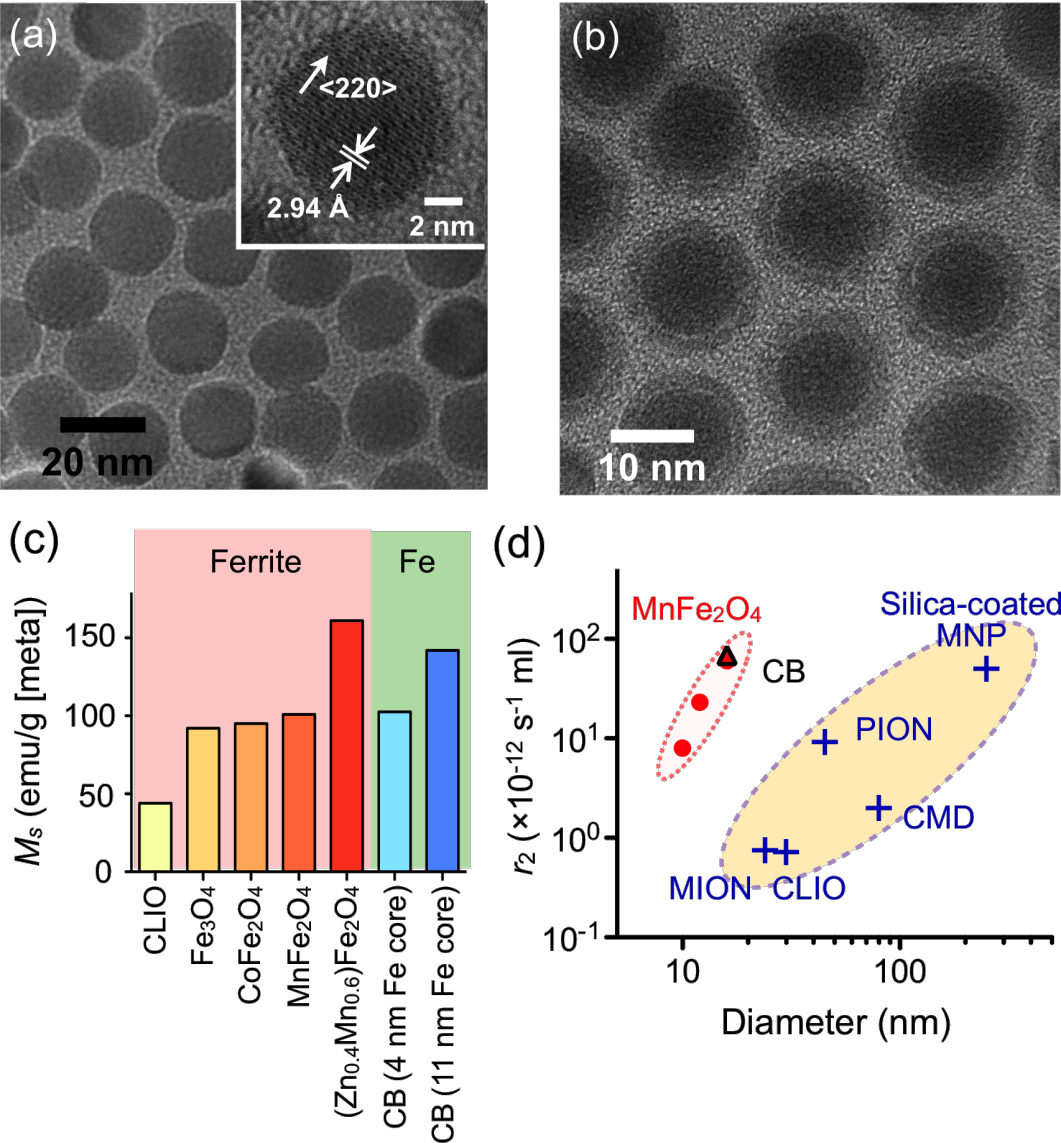
Higher *r*_2_-relaxivity MNPs developed to improve detection sensitivity of in vitro diagnostics. (a) Transmission electron micrograph (TEM) images of manganese-doped ferrite nanoparticles (MnFe_2_O_4_). These nanoparticles have narrow size distribution and high crystallinity, and were synthesized by a seed-growth method to produce 10, 12, 16, and 22 nm nanoparticles. (b) TEM image of elemental iron (Fe) core/ferrite shell magnetic nanoparticles (CB; cannonballs). These particles have a large Fe core (11 nm) passivated with a thin ferrite shell (2.5 nm), resulting in high particle relaxivity. (c) Summary of published saturated magnetizations (*M*_s_) for ferrite and Fe-based nanoparticles. Doped-ferrite and elemental Fe-based nanoparticles have improved *M_s_*. (d) Comparison of the size and the *r*_2_ relaxivity of various doped-ferrite and elemental Fe-based nanoparticles: CLIO, cross-linked iron oxide; MION, monocrystalline iron oxide; PION, polycrystalline iron oxide; and CMD, carboxymethyl dextran-coated MNP. (Adapted with permission from [15]. Copyright 2009 National Academy of Sciences, USA. Reproduced with permission from [16]. Copyright 2009 John Wiley and Sons, Inc.)

Both magnetic doping and sizing strategies were recently employed by our laboratory to produce MnFe_2_O_4_ nanoparticles with superior *r*_2_ relaxivity, for DMR biosensing applications [15]. These particles were synthesized by reacting iron(III) acetylacetonate [Fe(acac)_3_], manganese(II) acetylacetonate [Mn(acac)_2_] and 1,2-hexadecanediol at high temperature (300 °C). A seed-mediated growth approach was used to increase the size of the magnetic core from 10 nm to 12, 16, or 22 nm. MnFe_2_O_4_ nanoparticles with a diameter ≤16 nm were found to be highly monodisperse and superparamagnetic at 300 K (Figure 2a). The MNPs were subsequently rendered water-soluble using the small molecule, meso-2,3-dimercaptosuccinic acid (DMSA). DMSA has a terminal carboxylic acid group at one end which interacts directly with the magnetic core, and a sulfhydryl group at the other end which cross-links with other DMSA molecules to increase stability [27,33,47]. Due to DMSA’s small size, the hydrodynamic diameter of MnFe_2_O_4_ nanoparticles was found to be smaller than that of CLIO nanoparticles, despite their larger magnetic core. More importantly, these MnFe_2_O_4_ nanoparticles possessed superior relaxivities with *r*_2_ values as high as 420 s^−1^·mM^−1^[metal] (equal to 6 × 10^−11^ s^−1^·[particle/mL]^−1^), more than 8 times greater than CLIO nanoparticles in metal basis (50 s^−1^·mM^−1^[metal] or 0.7 × 10^−12^ s^−1^·[particle/mL]^−1^) [15].

### Elemental iron-based nanoparticles

The synthesis of elemental iron-based nanoparticles (i.e., with elemental iron rather than iron oxides) and their stable dispersion in aqueous media, has remained one of the most attractive goals in magnetic nanomaterial engineering. Elemental iron (Fe) has a higher magnetization than that of metal oxides, which consequently motivates the creation of Fe-core MNPs to achieve high *r*_2_ relaxivities [48–49]. Because the Fe cores are extremely reactive and subject to rapid oxidation, they need to be encased by a protective shell in order to maintain their magnetic properties. Recently, a 16 nm Fe-core/ferrite shell MNP, known as “cannonball”, was developed for DMR applications (Figure 2b) [16]. The cannonballs were synthesized by thermal decomposition of iron(0) pentacarbonyl [Fe(CO)_5_] to form the Fe core. A protective ferrite shell was formed by controlled oxidation with oxygen gas; this method resulted in a thinner shell than that previously produced by chemical oxidizers [49], and thus the nanoparticles retained a larger Fe core. The cannonballs were then coated with DMSA as described above. Because of their large Fe core, superparamagnetic cannonballs showed high magnetization (139 emu·g^−1^ [Fe]) when compared to other published Fe core–shell structures (Figure 2c). The relaxivity of cannonballs is similar to that of the MnFe_2_O_4_ nanoparticles (6 × 10^−11^ s^−1^·[particle/mL]^−1^), which is considerably higher than other commercially available or previously reported ferrite nanoparticles (Figure 2d).

### Bioorthogonal nanoparticle detection

**Figure 3:**
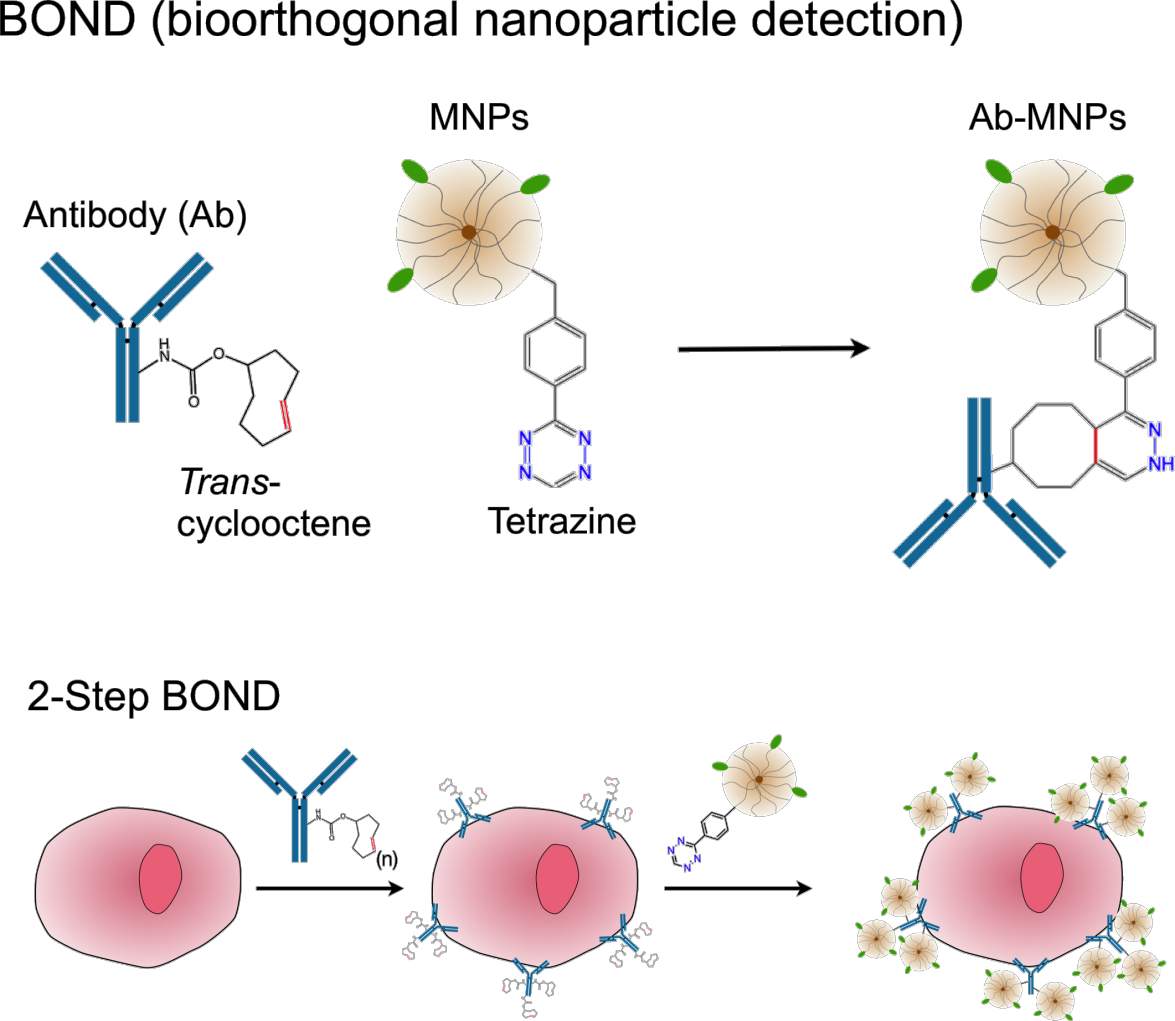
Bioorthogonal nanoparticle detection (BOND) strategy for DMR detection. The schematics show the conjugation chemistry between the antibody and the nanoparticle. This nanoparticle targeting platform uses a rapid, catalyst-free cycloaddition as the coupling mechanism. Antibodies against biomarkers were modified with *trans*-cyclooctene (TCO) and used as scaffolds to couple more tetrazine (Tz) modified nanoparticles onto live cells. The strategy is fast, specific, and amplifies biomarker signals. (Reproduced with permission from [53]. Copyright 2010 Nature Publishing Group.)

In addition to the previous strategies to improve the MNP core to enhance their relaxivities, surface modification of nanoparticles also improves their biosensing capabilities by amplifying their targeting valency for DMR applications. Bioorthogonal “click” chemistry has emerged as a novel method to label small molecules in complex biological media [50]. Most reported applications, however, rely on either the azide–alkyne cycloaddition, which requires a copper catalyst, or the strain-promoted azide–alkyne cycloaddition, which has relatively slow kinetics. Overcoming these limits, we have developed a new bioorthogonal chemistry based on the Diels–Alder cycloaddition between a 1,2,4,5-tetrazine (Tz) and a *trans*-cyclooctene (TCO). The reaction is fast, irreversible (covalent) and can be performed at room temperature without using a copper catalyst [51–52]. Recently, this chemistry has been successfully adapted to magnetic targeting, so as to improve nanoparticle binding efficiency and detection sensitivity. Termed ‘bioorthogonal nanoparticle detection’ (BOND), this technique provides a novel targeting platform in which Tz and TCO act as the coupling agents [53]. In a two-step labeling strategy (BOND-2; Figure 3), antibodies against biomarkers of interest are first modified with TCO, which is then used as a target to facilitate the coupling of Tz-modified nanoparticles onto mammalian cells. Because of the small size of the coupling reagents, their high multiplicity on antibodies/nanoparticles resulted in higher nanoparticle binding to cells. In comparison to alternative standard techniques, such as the avidin/biotin method, BOND-2 not only amplifies the biomarker signals but also significantly improves the detection sensitivity. Moreover, this platform is broadly-applicable and scalable for biomedical use. BOND-2 has already been successfully adapted for molecular profiling of cell samples by DMR [53], and has now established itself as a major targeting method in our laboratory. Table 1 lists a library of cellular makers tested with BOND-2 and DMR.

**Table 1:** List of extracellular and intracellular biomarkers tested with BOND-2 and DMR.

Tumors				Normal host cells

Extracellular		Intracellular		

A33	Glypican-3	α-fetoprotein	Cleaved PARP	anti-Fibroblast
B7H3	Hepsin	Cleaved CASP3	pH2AX	Calretinin A
B7-H4	HER2	CK5	phospho-EGFR	CD11b
CA125	HER3	CK7	phospho-p53	CD11c
CD133	Mesothelin	CK8	phospho-S6rp	CD14
CEA	MET	CK14	PSA	CD15
Claudin-1	Mucin1	CK18	PSMA	CD16
Claudin-3	Mucin16	CK19	s100A2	CD19
Claudin-7	Mucin18	CK20	s100A4	CD45
E-cadherin	P-cadherin	panCK	s100A6	CD56
EGFR	PCSA	EGFR-cytoplasmic	s100A11	CD68
EGFRv3	PSMA	gp100	s100B	CD56
EMMPRIN	PSAP	Ki-67	S6rp	CD68
EpCAM	TfR	MAGE-1	TTF-1	
FOLR1	TSPAN8	Melan-A	Tyrosinase	
FSH-R	uPAR	p53	Vimentin	
		PARP1	WT1	

## Miniaturized NMR systems

Nuclear magnetic resonance (NMR) can be detected with instruments such as clinical MRI scanners (routinely used for deep tissue whole body imaging), and NMR spectroscopy (used to study proteins and small molecules). Both of these techniques have been used to measure *T*_2_ relaxation time for DMR biosensing. However, because these conventional instruments are bulky and expensive, they remain as specialized equipment in hospitals and laboratories. Benchtop relaxometers, which operate at lower NMR frequencies (100 kHz–50 MHz) with a permanent low-field magnet (<1 T), provide a lower-cost alternative for DMR biosensing [13,40]. However, these systems lack the capability for performing multiplexed measurements, and require large sample volumes (>100 μL) to achieve accurate measurements.

### Chip-NMR biosensor

To overcome the limitations of conventional detectors and to address the need for fast, simple and high-throughput biosensing, our laboratory recently developed a chip-based microNMR (μNMR) device [14]. This miniaturized DMR device consists of an NMR probe containing microcoils for both radio-frequency (RF) excitation and NMR signal detection, on-board NMR electronics, a microfluidic network for sample handling, and a small permanent magnet for generating an external magnetic field.

**Figure 4:**
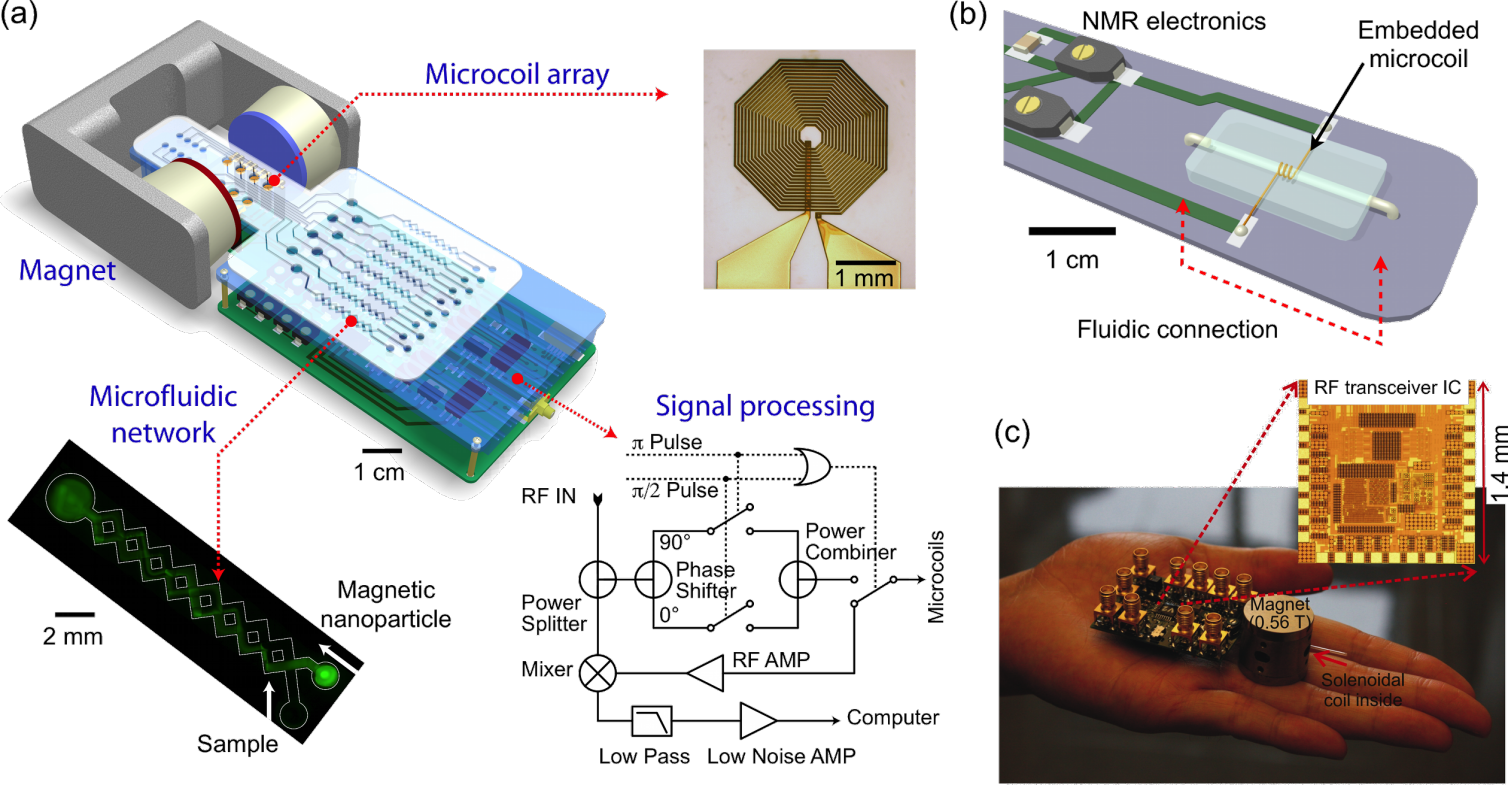
Miniaturized devices developed for DMR biosensing. (a) The first-generation miniaturized device to measure relaxation times of biological samples consists of an array of microcoils for NMR measurements, microfluidic networks for sample handling and mixing, miniaturized NMR electronics and a portable permanent magnet to generate a polarizing magnetic field. (b) The second-generation consists of a solenoidal coil embedded in a microfluidic device. As compared to the previous generation, this improved device has a higher filling factor, better signal-to-noise ratio, and reduced sample volume requirement to ~1 μL. (c) The latest 0.1 kg “palm” DMR system is 20× lighter and 30× smaller than previous generations. To achieve this significant size reduction, a small 0.56 T magnet was used. To compensate for the signal reduction from the smaller magnet, this device incorporates a new RF transceiver fully integrated in the 0.18 μm CMOS. (Reproduced with permission from [14]. Copyright 2008 Nature Publishing Group. Reproduced with permission from [15]. Copyright 2009 National Academy of Sciences, USA. Reproduced with permission from [19]. Copyright 2010 IEEE.)

The first μNMR prototype was designed with a 2 × 4 planar microcoil array that was lithographically patterned onto a glass substrate (Figure 4a) [14]. This array format enabled the performance of parallel measurements, and each microcoil held 5–10 μl of sample. In the second-generation μNMR, we changed our design to solenoidal coils [15–16], as such geometry provides higher signal-to-noise ratio (SNR) by producing more homogeneous radio-frequency magnetic fields for sample excitation. The SNR could be further increased by integrating the coil with a microfluidic channel (Figure 4b). The solenoidal coils were first wound around polyethylene tubes and subsequently immersed into a polymer (polydimethylsiloxan). Following polymer curing, the tubes were retracted to open up fluidic channels. The entire bore of the solenoid thus can be filled with sample to achieve maximal filling factor (≈1), the fraction of the coil volume occupied by the sample. Due to the larger cross-sectional area of the winding wires, the solenoidal coils also have smaller less electrical resistance than lithographically-patterned coils. With these advantages, the sample volume for DMR detection could be reduced by a factor of ~10 (to 1 µL) compared to the previous devices (~10 µL).

The microfluidic networks in the DMR system facilitate the handling of biological fluids, the effective mixing of MNPs with small sample volumes, and the distribution of small volumes to different coils for parallel sensing. The networks also serve to confine the samples to the most sensitive region of a given microcoil. Furthermore, a membrane filter can be inserted at the outlet of the solenoidal microcoil to retain large biological targets, whilst removing smaller contaminants such as unbound MNPs [16]. This configuration enables both the concentration of scant samples from large volumes, as well as the performance of on-chip washing steps.

The NMR electronics generate versatile RF pulse sequences to measure the longitudinal (*T*_1_) and transverse (*T*_2_) relaxation times, process raw NMR signals (amplification, frequency-conversion, filtering) for acquisition by a computer, and handle the multiplexed operation of an array of coils. In the first prototype, the NMR electronics was constructed as a tabletop system using discrete RF chips (e.g., AD9830 for RF generation and AD604 for NMR signal amplification; Analog Devices) and off-the-shelf RF components (e.g., ZAD-1 mixer, ZMSC-2 power splitter, and ZYSWA-2 RF switch; Mini-Circuits) [14]. In newer versions, these functionalities have been integrated onto a single CMOS IC chip [17,19]. This chip was designed to overcome the adverse effects associated with system miniaturization during NMR measurements, including 1) low NMR signals resulting from small sample volumes, and 2) fast signal decay due to field inhomogeneity produced by the portable magnet. These challenges were addressed by implementing low noise RF amplifiers with high voltage gain, and by developing an on-chip digital pulse generator for various pulse sequences. The latter enabled the accurate measurement of transverse (*T*_2_) relaxation times by generating Carr–Purcell–Meiboom–Gill (CPMG) sequences to compensate for the inhomogeneity of the polarizing magnetic field.

In the latest implementation, the entire DMR system was packaged as a handheld unit for portable operation (“palm” NMR system; Figure 4c) [19]. When benchmarked against conventional NMR systems, these miniaturized devices provided both superior detection sensitivities and capabilities for multiplexed measurements on small sample volumes. In view of such advantages, the miniaturized DMR technology is well suited for fast, simple and high-throughput analysis of scant biological samples within a point-of-care setting.

## DMR applications

DMR has been successfully applied to sensitively identify and quantify a wide range of biological targets including DNA/mRNA, proteins, enzyme activities, small molecules/drugs, bacteria, viruses and mammalian tumor cells, as summarized in Table 2. As described previously, the detection mode of DMR depends on the size of its target.

**Table 2:** Selected list of DMR assays developed to date^a^.

Type	Target	MNP Sensor (MNP core diameter <20 nm)	Reference

**DNA**	Telomeres	(CCCTAA)_3_-CLIO	[54]

**RNA**	GFP	CLIO-ATTTGCCGGTGT; TCAAGTCGCACA-CLIO	[13]

**Soluble Proteins**	Avidin	Biotin-CLIO	[14,55]
	GFP	Anti-GFP-CLIO	[13]
	β-HCG	Anti-HCG-CLIO	[56]
	Telomerase	Anti-telomerase-CLIO	[37]
	CA-125	Anti-CA125-CLIO	[14]
	VEGF	Anti-VEGF-CLIO	[14]
	α-fetoprotein	Anti-α-fetoprotein-CLIO	[14]

**Enzyme activities**	BamH1	CLIO-TTA-CGC-CTAGG-ATC-CTC; AAT-GCG-GGATCC-TAC-GAG-CLIO	[39]
	Methylase, Mbol, Dpnl	Methylated BamH1 CLIO	[39]
	Caspase-3	CLIO-Avidin-Biotin-GDEVDG-CLIO	[13]
	Renin	Biotin-IHPFHLVIHTK-Biotin; Avidin-CLIO	[57]
	Trypsin	Biotin-(G)_4_RRRR(G)_3_K-Biotin or Biotin-GPARLAIK-Biotin; Avidin-CLIO	[57]
	MMP-2	Biotin-GGPLGVRGK-Biotin; Avidin-CLIO	[57]
	Telomerase	CLIO-AATCCCAATCCC; AATCCCAATCCC-CLIO	[37,54]
	Peroxidases	Phenol-CLIO, tyrosines-CLIO	[41]

**Small molecules**	Drugs, enantiomers	D-Phenylalanine-CLIO	[58]
	Folate	Folate-CLIO	[59]
	Glucose	Concavalin-CLIO	[59]
	HA peptide	HA-CLIO	[59]
	Calcium	Calmodulin-CLIO; M13-CLIO or chelaters	[60–61]
	Influenza Tag peptide	Anti-Tag-CLIO	[62–63]

**Pathogens**	Herpes simplex virus	Anti-glycoproteinD(HSV-1)-CLIO; Anti-HSV1-CLIO	[40]
	Adenovirus-5	Anti-Adenovirus-5-CLIO	[40]
	*S. aureus*	Vancomycin-CLIO	[14]
	MTB/BCG	Anti-BCG-CLIO, Anti-BCG-Cannonball	[16]

**Cells (Extracellular and intracellular proteins)**	Tumor cell lines	Anti-Her2-CLIO, Anti-EGFR-CLIO, Anti-EpCAM-CLIO	[14]
	FNA (mouse xenograft)	Anti-Her2-MnFe_2_O_4_; Anti-EGFR-MnFe_2_O_4_, Anti-EpCAM-MnFe_2_O_4_	[15]
	Tumor cell lines (BOND amplification)	Anti-Her2-TCO, Anti-EGFR-TCO, Anti-EpCAM-TCO, Anti-Mucin1-TCO; Tz-CLIO; Many others, see Table 1	[53]

^a^BCG: Bacillus Calmette–Guérin; CLIO: cross-linked iron oxide; EGFR: epithelial growth factor receptor; EpCAM: epithelial cell adhesion molecule; FNA: fine needle aspirate; GFP: green fluorescent protein; HA peptide: hemagglutinin peptide; β-HCG: β-human chorionic gonadotropin; MMP-2: matrix metalloproteinase-2; MTB: mycobacterium tuberculosis; VEGF: vascular endothelial growth factor.

### Proteins

**Figure 5:**
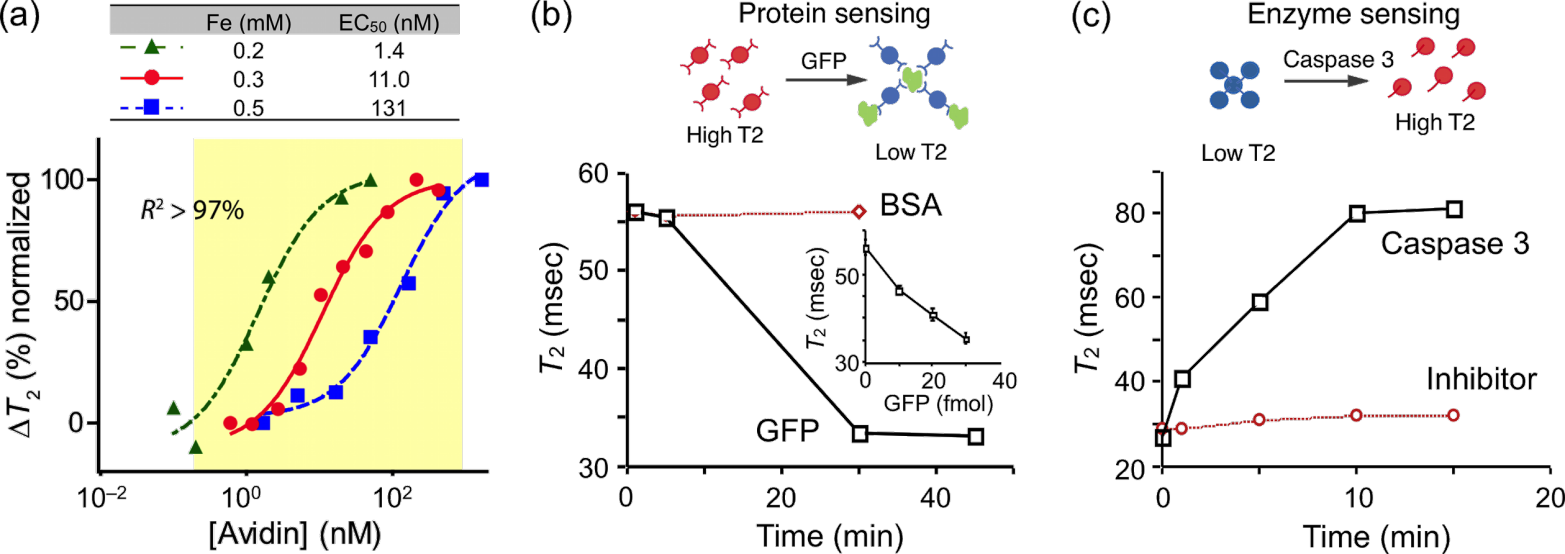
DMR detection of proteins and enzyme activities with MRSw sensors. (a) Detection of avidin. Biotinylated MNPs were incubated with different amounts of avidin to cause clustering of the nanoparticles. The *T*_2_ relaxation changes were dependent on avidin concentrations. (b) Detection of green fluorescent protein (GFP). MNPs conjugated with a polyclonal anti-GFP antibody were incubated with GFP or BSA as a control. *T*_2_ relaxation time decreased with increasing GFP concentrations; the detection limit was down to the low femtomolar range. (c) Detection of enzyme activity of caspase-3. MNPs were clustered with a peptide linker containing the sequence DEVD and were rapidly dissociated upon the activity of caspase-3. This dissociation was not observed when a specific caspase-3 inhibitor was added. The enzyme-dependent disassembly of the MNP clusters resulted in an increase in *T*_2_ relaxation time. (Reproduced with permission from [13–14]. Copyright 2002, 2008 Nature Publishing Group.)

The concept of forward MRSw sensing can be demonstrated with proteins, for example avidin, in model applications. In one early series of experiments, biotinylated MNPs were incubated with varying amounts of avidin [14]. As shown in Figure 5a, the binding of biotin to avidin resulted in clustering of MNPs and a concomitant avidin concentration-dependent change in *T*_2_. By varying the concentration of MNPs, four orders of dynamic ranges were achieved, indicating that the system has a robust working range. Likewise, specific antibodies can also be used to perform MRSw on target protein molecules. As the second proof-of-principle analysis, green fluorescent protein (GFP)-sensitive nanoparticles were prepared by conjugating CLIO nanoparticles with anti-GFP polyclonal antibodies [13]. Using this system, GFP was rapidly and sensitivity detected in a dose-dependent manner while the addition of bovine serum albumin (BSA) protein as a control did not elicit any change in *T*_2_ (Figure 5b). More recently, MRSw biosensors, capable of detecting soluble tumor biomarker proteins (such as CA-125, VEGF, and α-fetoprotein) were described, and used for parallel detection of multiple markers in blood samples with the μNMR device [14]. Finally, using the BOND-2 method, many other cancer proteins have been detected (Table 1).

### Enzyme activities

The reverse MRSw strategy has been widely applied to the detection of enzymatic activities. Reverse sensors have been designed to detect and quantify proteases [38,64], endonucleases and methylases [39]. In these assays, the enzyme activity disassembles pre-formed clusters of MNPs; this disintegration translates the enzymatic activity into a detectable *T*_2_ signal. In the first demonstration of this strategy, MNP aggregates were formed with the peptide sequence biotin-GDEVDGC. This sequence served as a linker, binding both an avidin-conjugated CLIO population (via the biotin/avidin interaction) as well as a second CLIO population (via the thiol provided by the terminal cysteine on the peptide) [13]. The subsequent addition of caspase-3 disassembled the aggregates by cleaving within the DEVD site, which led to a corresponding increase in *T*_2_ relaxation time (Figure 5c). This dissociation was not observed when a specific caspase-3 inhibitor was added. A similar reverse switching strategy has been used to detect trypsin, renin, and matrix metalloproteinase 2 activities [57].

Forward MRSw assays on enzymatic activities have also been demonstrated via the assembly of nanoparticle biosensors (as a result of enzymatic reactions). For example, specific MNPs have been designed to assess human telomerase (hTERT) activity by hybridizing with the 30-base pair telomeric repeat sequences produced by hTERT activity [54]. More recently, myeloperoxidase (MPO) sensors were generated by attaching phenol-containing molecules, such as dopamine or serotonin, to CLIO nanoparticles [41]. In the presence of peroxidase activity, tyroxyl radicals were formed to cross-link the nanoparticles. Using the same assay configuration, leukocyte-derived MPO has been shown to play a critical role in the pathogenesis of atherosclerotic plaques [65].

### Bacteria

**Figure 6:**
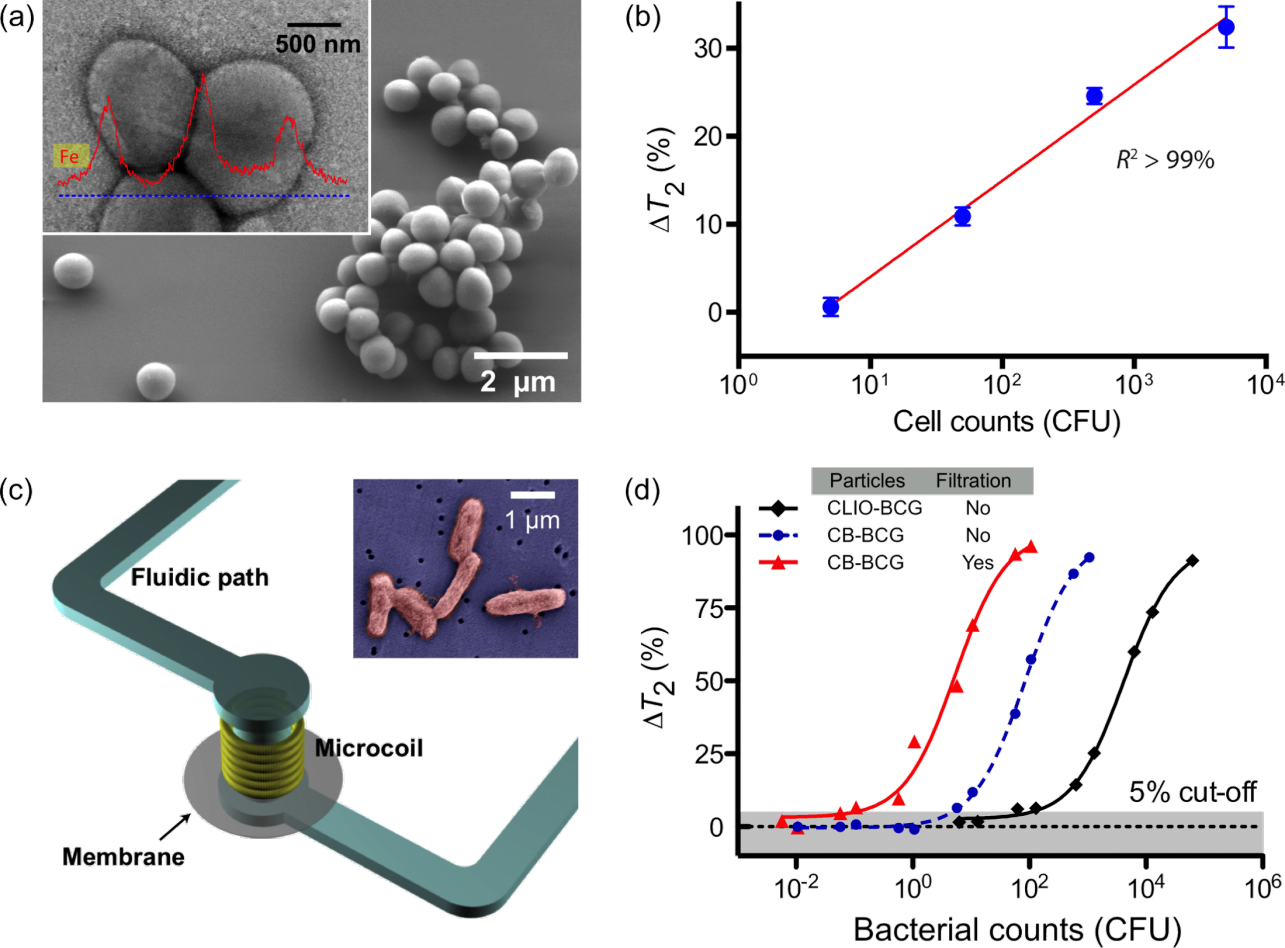
DMR detection of bacteria by tagging the bacterial samples with MNPs. (a) Scanning electron micrograph of *S. aureus.* Inset shows TEM of *S. aureus* targeted by CLIO conjugated with vancomycin. MNPs formed dense clusters on the bacterial wall. Elemental analysis by energy dispersive X-ray spectrometry further confirmed the binding of nanoparticles to the bacteria. (b) Changes to *T*_2_ with varying number of *S. aureus.* The DMR system had a detection sensitivity of a few colony-forming units (CFUs) per microliter, with dynamic ranges over three orders of magnitude. (c) NMR-filter system for bacterial concentration and detection. It consists of a microcoil and a membrane filter integrated with a microfluidic channel. The membrane filter concentrates bacteria inside the NMR detection chamber to achieve high-detection sensitivity. Inset shows bacteria (Bacillus Calmette–Guérin, BCG) captured on the membrane filter after filtration. (d) Changes to *T*_2_ with varying BCG bacterial counts. Detection limit was approximately 100 CFUs with CLIO nanoparticles and 6 CFUs with higher relaxivity cannonballs. Detection sensitivity was further increased to ~1 CFU using the built-in filtration. CLIO, cross-linked iron oxide; CB, cannonball (Fe@ferrite) MNP. (Reproduced with permission from [14]. Copyright 2008 Nature Publishing Group. Reproduced with permission from [16]. Copyright 2009 John Wiley and Sons, Inc.)

Detection and quantification of large pathogens have been successfully demonstrated using the DMR platform, primarily through magnetic tagging of targets. For example, detection of the bacterium *Staphylococcus aureus* was recently reported with the μNMR device. *S. aureus* were initially incubated with MNPs derivatized with vancomycin, a drug which binds to D-alanyl–D-alanine moieties in the bacterial cell wall to form dense clusters (Figure 6a) [14]. On account of the low sample volume required by the μNMR device, this first proof-of-concept analysis demonstrated a detection sensitivity of only a few colony-forming units (CFUs) per microliter sample (Figure 6b).

More recently, tuberculosis (TB) bacteria have been detected using DMR. In one study, the highly magnetic Fe-core/ferrite shell nanoparticles (CB; cannonballs) were used in combination with the second generation DMR device [16]. To evaluate the clinical utility of the DMR platform for TB detection, Bacillus Calmette-Guérin (BCG), used as a surrogate for *Mycobacterium tuberculosis*, was spiked into sputum samples. Following liquefaction, the biological samples were incubated with cannonballs conjugated to an anti-BCG monoclonal antibody. Unbound MNPs were then removed via a built-in membrane filter, embedded within the device (Figure 6c). This membrane (~100 nm size cut-off) not only removed excess unbound MNPs but also retained the BCG bacteria; thus was effective for both concentrating scant bacteria and removing background signal. In comparison to standard TB diagnostics, which involve time-consuming culture and acid-fast bacilli (AFB) smear microscopy, the DMR diagnostic technology showed unprecedented detection sensitivity and speed: as few as 20 CFUs could be detected in 1 ml of sputum sample, in less than 30 minutes (Figure 6d). Currently, this detection technology is being adapted to detect infectious pathogens in clinical sputum samples.

### Tumor cells

Sensitive detection and rapid profiling of tumor cell surface markers in unprocessed biological samples will undoubtedly have a significant impact on both the life sciences and clinical practice. DMR molecular profiling of Her2/*neu*, EGFR, and CD326 (EpCAM) cancer markers on mammalian cells was first demonstrated using the first-generation DMR device [14]. In these early experiments, CLIO nanoparticles were directly conjugated to monoclonal antibodies. More recently, the use of BOND-2 strategy has further advanced DMR profiling capabilities (Table 1). Cancer cells were targeted with CLIO nanoparticles via BOND-2. At a low cell count (~1000 cells per sample), parallel DMR measurements could be performed rapidly [53]. As a universal labeling approach, BOND-2 simplifies the preparation of the targeted MNPs for multiplexing and amplifies nanoparticle binding to cells.

**Figure 7:**
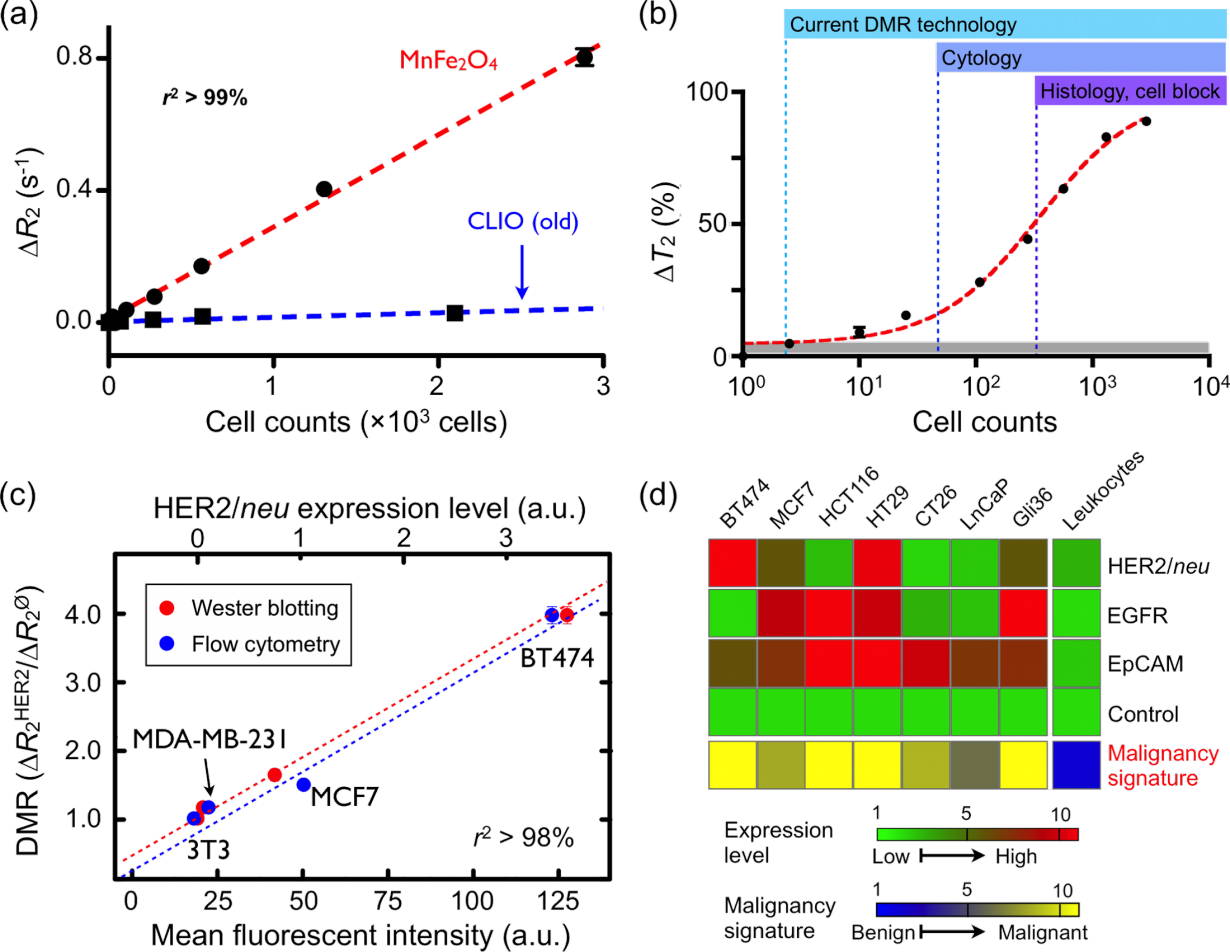
Tumor cell detection and profiling with the µNMR device. (a) Human breast cancer cells (BT474) were labeled with anti-Her2 CLIO and MnFe_2_O_4_ nanoparticles. The change in *R*_2_ (*R*_2_ =1/*T*_2_) varied linearly with cell counts, and the detection sensitivity was 10× better using the more magnetic MnFe_2_O_4_ nanoparticles. (b) The detection sensitivity was approximately two cells (in 1 µL sample volume) with the improved µNMR device (Figure 4b) and the highly magnetic MnFe_2_O_4_ nanoparticles, making this detection platform superior to current clinical methods (cytology and histology). (c) DMR measurements correlated well with standard molecular analyses, such as flow cytometry and Western blot, but required substantially fewer cells. (d) Molecular profiling of fine-needle aspirates of mouse tumor xenografts. Three cancer markers (Her2/*neu*, EGFR, EpCAM) were profiled to increase the accuracy of diagnosis. Unmodified nanoparticles were used as a control to estimate cell concentration based on non-specific phagocytosis. (Reproduced with permission from Ref [15]. Copyright 2009 National Academy of Sciences, USA.)

Using the μNMR device with a solenoidal coil and the highly magnetic MnFe_2_O_4_ nanoparticles, detection sensitivity for cell sensing was remarkably improved (Figure 7a) [15]. Notably, as shown in Figure 7b, the detection threshold was reduced to approximately single-cell level, far surpassing the sensitivity seen in either previous DMR experiments or other conventional clinical methods. There was also a good correlation between DMR measurements and those obtained with flow cytometry and Western blot analysis (Figure 7c). Importantly, the DMR detection platform not only required far fewer cells than either of the alternative approaches, but also produced results in a fraction of the time (<15 minutes). The DMR platform has since been shown to be adaptable to rapid multi-target detection, where putative cancer cells can be profiled for multiple biomarkers; DMR is ideally suited to this use since it can perform measurements on a few cells in small sample volumes and in a multiplexed manner. Fine-needle aspirate biopsies from a panel of mouse xenograft tumors have already been successfully analyzed for Her2/*neu*, EGFR, and EpCAM expression. Furthermore, the multiple-marker targeting strategy has been shown to significantly improve the accuracy for correctly diagnosing cancer cells as malignant (Figure 7d). These, in addition to other advanced refinements to DMR sensing, are currently being applied to clinical trials of cancer cell profiling.

## Conclusion

DMR represents a powerful combination of several cutting-edge technologies, namely nanomaterials, bioconjugation chemistry and microfabrication. As a novel technique, DMR offers a number of unique advantages, such as high detection sensitivity, rapid target measurement from minimal sample volumes, and the ability to profile a wide range of targets in a multiplexed manner. With new developments such as the advent of chip-based μNMR devices, optimized magnetic nanomaterials and advanced conjugation techniques, DMR shows potential as a robust and easy-to-use sensor system with significantly improved sensitivity and accuracy. Thus, it is likely that this technology will have broad applications in biomedicine, as well as clinical utility in point-of-care settings.
